# Abnormal topological organization of functional brain networks in the patients with anterior segment ischemic optic neuropathy

**DOI:** 10.3389/fnins.2024.1458897

**Published:** 2024-11-22

**Authors:** Fei Chen, Xin-Miao Wang, Xin Huang

**Affiliations:** ^1^Department of Opthalmology, The First Affiliated Hospital of Zhengzhou University, Zhengzhou, Henan, China; ^2^School of Ophthalmology and Optometry, Jiangxi Medical College, Nanchang University, Nanchang, Jiangxi, China; ^3^Department of Ophthalmology, Jiangxi Provincial People’s Hospital, The First Affiliated Hospital of Nanchang Medical College, Nanchang, Jiangxi, China

**Keywords:** graph theory, brain network, functional connectome, resting-state functional magnetic resonance imaging, AION

## Abstract

**Objective:**

An increasing amount of neuroimaging evidence indicates that patients with anterior segment ischemic optic neuropathy (AION) exhibit abnormal brain function and structural architecture. Some studies have shown that there are abnormal functional and structural changes in the brain visual area of AION patients. Nevertheless, the alterations in the topological properties of brain functional connectivity among patients with AION remain unclear. This study aimed to investigate the topological organization of brain functional connectivity in a group of AION patients using graph theory methods.

**Methods:**

Resting-state magnetic resonance imaging was conducted on 30 AION patients and 24 healthy controls (HCs) matched for age, gender, and education level. For each participant, a high-resolution brain functional network was constructed using time series correlation and quantified through graph theory analysis.

**Results:**

Both the AION and HC groups presented high-efficiency small-world networks in their brain functional networks. In comparison to the HCs, the AION group exhibited notable reductions in clustering coefficient (Cp) and local efficiency (Eloc). Specifically, significant decreases in Nodal local efficiency were observed in the right Amygdala of the AION group. Moreover, the NBS method detected a significantly modified network (15 nodes, 15 connections) in the AION group compared to the HCs (*p* < 0.05).

**Conclusion:**

Patients with AION exhibited topological abnormalities in the human brain connectivity group. Particularly, there was a decrease in Cp and Eloc in the AION group compared to the HC group. The anomalous node centers and functional connections in AION patients were predominantly situated in the prefrontal lobe, temporal lobe, and parietal lobe. These discoveries offer valuable perspectives into the neural mechanisms associated with visual loss, disrupted emotion regulation, and cognitive impairments in individuals with AION.

## Introduction

Ischemic optic neuropathy (ION) refers to optic nerve damage resulting from a temporary or permanent disruption of blood supply to any part of the optic nerve (Hayreh, 2009). One of the most prevalent and visually debilitating conditions in the middle-aged and elderly population is Ischemic Optic Neuropathy (ION). This condition manifests in two distinct types: anterior (AION) and posterior (PION). AION affects the optic nerve head (ONH), while PION impacts the remainder of the optic nerve. The pathogenesis of AION and PION vastly differs. Anterior Ischemic Optic Neuropathy (AION) is characterized by acute ischemia and hypoxia of the optic nerve due to blockage of the posterior ciliary artery supplying the optic nerve. This blockage leads to optic disc edema and visual field defects. AION stands as the most prevalent acute optic neuropathy in individuals aged 50 and above (Ing et al., 2020). Ischemic optic neuropathy can affect one eye or both eyes. Without prompt treatment or in cases of misdiagnosis, recovery of visual acuity and visual field can be challenging. The incidence of this condition has notably risen in recent years (Kellett and Madonna, 1997). In contrast, PION does not have a specific location within the posterior segment of the optic nerve and is not associated with a distinct arterial ischemic disorder. Clinically, AION presents in two forms: arteritic AION (A-AION) caused by giant cell arteritis, and non-arteritic AION (NA-AION). NA-AION, the more common variant, is a prevalent and visually debilitating condition affecting the middle-aged and elderly population, with the potential to occur bilaterally. Previous studies (Zhao et al., 2021; Wang et al., 2024) have demonstrated that AION is not only an ocular disease but also a neuro-ophthalmological condition.

Overall, limited research has been conducted on the structural and functional plasticity of the brain in AION cases, as conventional magnetic resonance imaging (MRI) offers limited insights into the lesion. Functional magnetic resonance imaging (fMRI), a non-invasive neuroimaging technique, offers the opportunity to investigate neural plasticity in NAION cases. fMRI has been extensively utilized to investigate changes in brain function in various eye-related diseases (Burton et al., 2004; Wang et al., 2008; Shao et al., 2015; Li et al., 2016; van Kemenade et al., 2017; Huang et al., 2018; Min et al., 2018; Xu et al., 2019). Furthermore, certain fMRI investigations have verified the existence of atypical spontaneous brain activity in patients with AION. Various rs-fMRI studies focusing on AION have demonstrated irregularities in spontaneous brain activity (Aguirregomozcorta et al., 2011; Guo et al., 2020; Guo et al., 2019). An earlier fMRI investigation revealed a decrease in activation in the bilateral occipital cortex when the affected eye was stimulated in patients with AION compared to healthy controls (Aguirregomozcorta et al., 2011). In addition, another study has shown that AION patients have local neuronal activity abnormalities in the right lingual gyrus, left putamen/lentiform nucleus, and left superior parietal lobule (Guo et al., 2020). In conclusion, the current study has found that AION patients have abnormal changes in the occipital lobe, lenticular nucleus and parietal lobe. However, our brain is a network of numerous brain regions where information is continuously processed and transmitted between structurally and functionally connected areas. Studying the human brain as a network of interacting regions can provide new insights into large-scale neurotransmission in the brain (Ke et al., 2024). Research has found that the development of most neurological and psychiatric disorders is associated with impaired interconnections between neurons and synapses (Kenney and Frankland, 2014). Although previous studies have suggested changes in the structure and function of local brain regions in AION patients, this does not reflect the ability of the entire brain of AION patients to process information. Therefore, the study of the functional brain network of AION patients helps us to understand the neural mechanism of AION more deeply, and provides a scientific basis for the prevention, diagnosis and treatment of AION.

Graph theoretic analysis is a mathematical algorithm utilized for the analysis of intricate brain networks. It quantitatively delineates the organizational structures of brain networks, offering a novel approach to measure brain organization and methodically characterize the topological properties of these networks (Bullmore and Sporns, 2009; He and Evans, 2010; Lo et al., 2011). During the investigation of brain structural networks using graph theoretic analysis, the brain network comprises nodes representing anatomical brain regions and edges representing fiber bundles connecting these regions. Through quantitative analysis of global attributes, node attributes, the organization of rich club, and other network characteristics, we can gain insights into the operational dynamics and information transmission properties of the brain (Bullmore and Sporns, 2009; Salvador et al., 2005; He et al., 2007; Tononi et al., 1994). Currently, complex network analysis utilizing graph theory has been extensively employed in the analysis of various neurological diseases (Mihaescu et al., 2021; Song et al., 2022; Tan et al., 2019). Neuroimaging studies have uncovered that “small-world” networks represent a distinct topological feature of the human brain (Bassett et al., 2006; Achard et al., 2006; Bassett and Bullmore, 2017). Watts et al. first introduced a mathematical model of a small-world network, which acts as an intermediary state between a regular network and a random network. Small-world networks exhibit high clustering and short path lengths, facilitating efficient and rapid information transmission at a minimal “wiring cost” (Watts and Strogatz, 1998; Sporns and Zwi, 2004; Achard and Bullmore, 2007). Graph theoretic methods have been utilized to explore the topological arrangement of structural networks in AION patients. Nonetheless, previous studies focused solely on structural networks, leaving the topological characteristics of functional brain networks in AION patients largely unexplored.

In this study, our objective was to evaluate brain network changes at both global and local levels in AION patients and investigate the correlation between these topological changes and clinical parameters to validate our hypothesis that AION patients would present with aberrant brain functional networks. We utilized resting-state functional magnetic resonance imaging (rs-fMRI) to construct functional brain connectomes for individuals with Anterior Ischemic Optic Neuropathy (AION) and healthy controls (HCs). Subsequently, we employed graph-theoretic methods to explore the modifications in the topological properties of functional connectomes in AION patients. Initially, we calculated and compared the global network metrics between the AION patients and the HC group. We then scrutinized the disparities in the node properties of the functional networks across the two cohorts. Finally, we investigated the abnormal functional connectivity in AION patients using the Network-based Statistic (NBS) method. Our study explored the effect of AION on brain function from the perspective of brain network, which provided a reference for exploring the mechanism of brain network injury in AION patients, and also provided important biological markers for the treatment and intervention of AION in the future.

## Materials and methods

### Subjects

In this study, a total of 54 participants took part, including 30 individuals diagnosed with Anterior Segment Ischemic Optic Neuropathy (AION) and 24 healthy controls (HCs). For the homogeneity of the two groups of samples, the study included the matching of gender, age and education level of the subjects. The research protocol followed the guidelines of the Declaration of Helsinki and received approval from the Research Ethics Committee of The First Affiliated Hospital of Zhengzhou University. Before participating in the study, all participants were briefed on the study’s objectives, methods, and potential risks, and they provided written informed consent to partake in the research.

Inclusion Criteria: Participants had to meet the diagnostic criteria for AION, which included sudden visual dysfunction, minimal eye pain, nerve bundle visual field defects, non-congestive optic disc edema, and limited peripapillary hemorrhage. Furthermore, they demonstrated delayed filling of the optic disc during fluorescein fundus angiography compared to the visual field, along with telangiectasia and capillary leakage on the surface of some filling defects.

During the same time frame, 24 healthy volunteers with similar educational backgrounds were selected as healthy controls (HC). The inclusion criteria for the HC group were as follows: (1) absence of ocular diseases; (2) unaided or corrected visual acuity >1.0; (3) no neurological or other comorbidities; and (4) absence of contraindications for magnetic resonance imaging. Patients with optic nerve atrophy, optic neuritis, diabetic retinopathy, retinal detachment, central retinal artery occlusion, panuveitis, glaucoma, or other retinal, optic nerve, and choroidal involvements were excluded from the study.

### MRI data acquisition

Data acquisition was performed in the MRI examination room of The First Affiliated Hospital of Zhengzhou University, all subjects were examined by using a 3.0-T MR imaging system (MAGNETOMPrisma; Siemens Healthcare, Erlangen, Germany) with a 64-channel head coil. The scanning parameters of bold were as follows: repetition time (TR) = 1,000 ms, echo time (TE) = 30 ms, flip angle (FA) = 70°, field of view (FOV) =220 × 220 mm^2^, slice thickness = 2.2 mm, slice number = 52, volumes = 400, voxel size = 2.0 × 2.0 × 2.2 mm^3^.

The subject was lying supine, and to minimize motion artifacts during image acquisition, the subject’s head was immobilized with padding and earplugs were worn to reduce scanning noise. During the MRI scan, all participants were asked to remain still during the MRI scan, to relax with their eyes closed, to maintain a steady breathing rhythm, and not to focus on any particular thoughts.

### Data preprocessing

DPABI: Data Processing and Analysis for (Resting-State) Brain Imaging in MATLAB 2013a (Myers Walker, Natick, MA, United States) were used in the study. The fMRI data was processed as outlined below: (1) The initial 10 volumes were discarded to eliminate the unstable signal from the NMR machine scanning at the beginning of the scan. (2) Slice time correction was performed. (3) Head movement correction was conducted. During magnetic resonance scanning, subjects may experience head movements due to factors such as mouth breathing or swallowing. Given the prolonged scanning duration and sequential interlayer scanning, these movements can interfere with the accuracy of the BOLD signal. Hence, it was essential to correct head movements to mitigate the impact of minor head motions on the magnetic resonance results. Rigid body transformation was used to transform 24 head motion parameters. Based on the estimation of motion calibration, the data of subjects with maximum head motion translation >1.5 mm and maximum rotation angle >1.5° were eliminated. According to the method of Van Dijk et al. (2012), the voxel frame displacement parameter (FD) was calculated, and there was no significant difference in FD between the two groups. (4) Image standardized registration. First, we use the 3D structure image of each subject to segment by (DARTEL) method to generate the anatomical template of each subject to register the functional image (Goto et al., 2013). Subsequently, the functional images of each subject were aligned with the MNI standard space using a voxel size of 3 × 3 × 3 mm^3^ to minimize inaccuracies or displacements of functional regions. Spatial smoothing was conducted by applying a Gaussian kernel with a half-width of 6 × 6 × 6 mm to enhance the signal-to-noise ratio of the images. Linear detrending was performed on data with linear trends, followed by linear regression analysis to account for several covariates, including Friston 24-parameter parameters and mean framewise displacement.[FD], global brain signal, and more (Yan et al., 2013; Fox et al., 2009). Lastly, a temporal band-pass filter (0.01–0.08 Hz) was applied to attenuate the effects of low-and high-frequency physiological noise.

**Figure 1:**
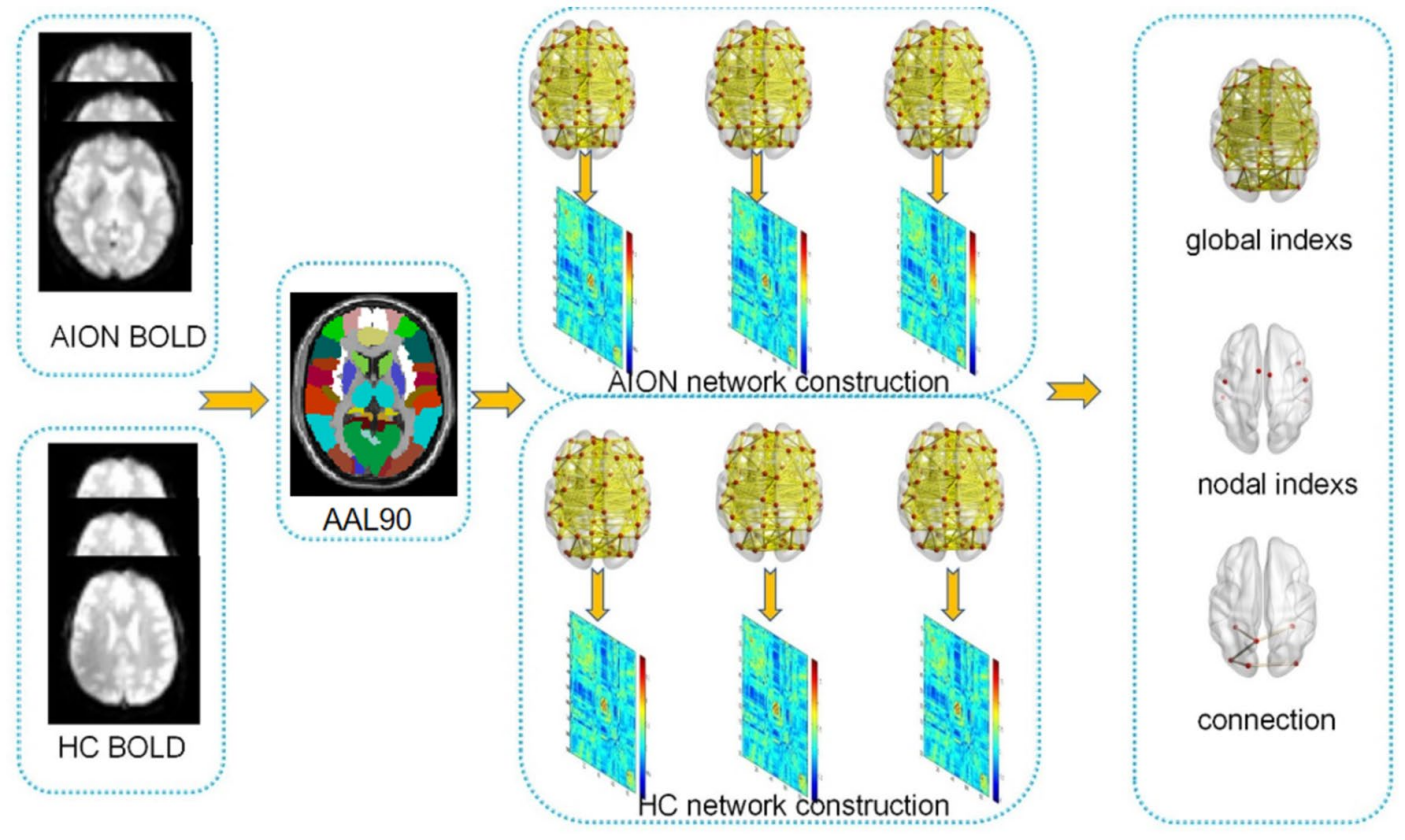
Presents a flowchart outlining the network construction and analysis using graph theory.

An overview of the data collection and analytical procedures is depicted in Figure 1.

### Functional network construction

#### Node and edge definitions

The network was constructed by using the graph theoretical network analysis.

toolbox GRETNA.^1^ In this study, we used the automated anatomical labeling (AAL) atlas (Bullmore and Sporns, 2009) o divide the cerebral cortex into 90 cortical regions (45 per hemisphere, see Table 1 for details), and each brain region representing a node in the cortical network. The process is as follows: To define the edges of the network, the average time series for each brain region was extracted. Pearson’s correlation coefficients were calculated between the regional mean time series of all possible pairs of the 90 brain regions to establish the network edges, resulting in a 90 × 90 Pearson’s correlation matrix. Further, Fisher’s r-to-z transformation is applied to correlation matrices (Liu et al., 2017), converting each correlation matrix into a binarized matrix with sparsity values. When the Pearson correlation coefficient is greater than the sparse value, it is considered that there is a corresponding edge in the brain network (Lu et al., 2017; Wang et al., 2020).

**Table 1:** The regions are identified based on a predefined template of the automated anatomical labeling atlas.

Index	Regions	Abbreviation
(1,2)	Precental gyrus	PreCG
(3,4)	Superior frontal gyrus, dorsolateral	SFGdor
(5,6)	Superior frontal gyrus, orbital part	ORBsup
(7,8)	Middle frontal gyrus	MFG
(9,10)	Middle frontal gyrus, orbital part	ORBmid
(11,12)	Inferior frontal gyrus, opercular part	IFGoperc
(13,14)	Inferior frontal gyrus, triangular part	IFGtriang
(15,16)	Inferior frontal gyrus, orbital part	ORBinf
(17,18)	Rolandic operculum	ROL
(19,20)	Supplementary motor area	SMA
(21,22)	Olfactory cortex	OLF
(23,24)	Superior frontal gyrus, media	SFGmed
(25,26)	Superior frontal gyrus, medial orbital	ORBsupmed
(27,28)	Gyrus rectus	REC
(29,30)	Insula	INS
(31,32)	Anterior cingulate and paracingulate gyri	ACG
(33,34)	Median cingulate and paracingulate gyri	DCG
(35,36)	Posterior cingulate gyrus	PCG
(37,38)	Hippocampus	HIP
(39,40)	Parahippocampal gyrus	PHG
(41,42)	Amygdala	AMYG
(43,44)	Calcarine fissure and surrounding cortex	CAL
(45,46)	Cuneus	CUN
(47,48)	Lingual gyrus	LING
(49,50)	Superior occipital gyrus	SOG
(51,52)	Middle occipital gyrus	MOG
(53,54)	Inferior occipital gyrus	IOG
(55,56)	Fusiform gyrus	FFG
(57,58)	Postcentral gyrus	PoCG
(59,60)	Superior parietal gyrus	SPG
(61,62)	Inferior parietal, but supramarginal and angular gyri	IPL
(63,64)	Supramarginal gyrus	SMG
(65,66)	Angular gyrus	AMG
(67,68)	Precuneus	PCUN
(69,70)	Paracentral lobule	PCL
(71,72)	Caudate nucleus	CAU
(73,74)	Lenticular nucleus, putamen	PUT
(75,76)	Lenticular nucleus, pallidum	PAL
(77,78)	Thalamus	THA
(79,80)	Heschl gyrus	HES
(81,82)	Superior temporal gyrus	STG
(83,84)	Temporal pole: superior temporal gyrus	TPOsup
(85,86)	Middle temporal gyrus	MTG
(87,88)	Temporal pole: middle temporal gyrus	TPOmid
(89,90)	Inferior temporal gyrus	ITG

### Network analysis

#### Threshold selection

To maintain uniform correlation levels across groups, a diverse range of sparsity (Sp) thresholds (S) were utilized for all correlation matrices. Sp corresponds to the proportion of actual edges to the maximum potential edges in a network, ensuring a consistent number of edges in all networks and reducing discrepancies in overall correlation strength between groups. This study employed an extensive range of Sp levels (ranging from 0.032 to 0.492, with intervals of 0.01). The area under the curve (AUC) for each network metric was calculated across the Sp range from S1 to Sn, with an interval of ΔS, to reveal variations in the topological characteristics of the brain networks. This aggregated AUC metric is adept at identifying alterations in the topological structure of the brain’s functional connectome.

#### Global and nodal network organization

Graph theoretical analysis of network connectivity and topology was performed using the GRETNA software (Wang et al., 2015) based on the Matlab platform and visualized using BrainNet Viewer software (Xia et al., 2013). The topological properties of the brain’s functional networks were computed at both global and local levels for each threshold. The global metrics included small-world parameters (Watts and Strogatz, 1998),encompassing the clustering coefficient (Cp), characteristic path length (Lp), *γ*, normalized characteristic path length (*λ*), as well as scalar small-worldness (*σ*); and network efficiency (Latora and Marchiori, 2001), involving Eglob and Eloc. The specific definitions of small-world and network efficiency properties, namely Cp, Lp, *γ*, *λ*, *σ*, Eglob, and Eloc, are detailed in Table 2. A small-world network with significantly higher Cp and similar Lp compared to random networks (1,000 matched random networks) was determined based on the following criteria: γ = Cpreal/Cprand >1 and λ = Lpreal/Lprand ≈ 1, which together form the small-worldness equation, σ = γ/λ > 1. Nodal metrics of functional networks are comprised of nodal degree, nodal efficiency, and nodal betweenness.

**Table 2:** Descriptions of the network metrics examined in this study.

Attribute	Character	Description
Clustering coefficient Characteristic path length Gamma Lambda Sigma Global efficiency Local efficiency	Cp Lp γ λ σ Eglob Eloc	The extent of local interconnectivity or cliquishness of a network The extent of overall communication efficiency of a network The deviation of Cp of a network from those of surrogate random networks The deviation of Lp of a network from those of surrogate random networks The small-worldness indicating the extent of a network between randomness and order The ability of a network to transmit information at the global level The ability of a network to transmit information at the local level
Betweenness Degree Efficiency	bi ki ei	The influence that one node has over the flow of information between all other nodes in the network The number of edges linked to a node The ability of a node to propagate information with the other nodes in a network

Cp, clustering coefficient; Lp, characteristic path length; γ, normalized clustering coefficient; λ, normalized characteristic path length; σ, scalar smallworldness; Eglob, global efficiency; Eloc, local efficiency.

### Statistical analysis

The clinical characteristics of the two groups were analyzed using the Statistical Package for Social Sciences (SPSS Version 26.0). Gender comparisons were performed with the chi-square test, while age, education, and best-corrected visual acuity (BCVA) were compared using the two-sample *t*-test. Statistical significance was set at a *p*-value <0.05.

The two-sample *t*-test was employed to evaluate group variances in the six global network parameters (*p* < 0.05) and the three regional nodal parameters (*p* < 0.05, Bonferroni-corrected). The area under the curve (AUC) for each metric was computed for statistical comparison across the Sp range (0.032 < Sp < 0.492, with intervals of 0.01). Covariates included in the analysis were age, sex, educational level, and mean FD.

To identify specific pairs of brain regions exhibiting altered functional connectivity in AION patients, we pinpointed region pairs that illustrated between-group differences in nodal characteristics. We then employed the network-based statistics (NBS) method^4^ (Zalesky et al., 2010).

## Results

### Demographic and clinical characteristics

Significant differences were observed in best-corrected visual acuity (*p* < 0.001) between the two groups. However, there were no significant differences in sex, age, index between the two groups. Further details are provided in Table 3.

**Table 3:** Demographics and clinical characteristics between two groups.

Condition	AION group	HC group	*T*-value	*P*-value
Gender (male/female)	17/13	9/15	1.977	0.160
Age (years)	54.56 ± 9.91	42.45 ± 10.18	4.408	<0.001*
BCVA-OD	0.28 ± 0.27	1.25 ± 0.25	−13.173	<0.001*
BCVA-OS	0.56 ± 0.39	1.25 ± 0.25	−7.686	<0.001*

Chi-square test for gender. Independent *t*-test was used for other normally distributed continuous data. Data are presented as mean ± standard deviation. AION, Anterior segment ischemic optic neuropathy; HC, healthy control; BCVA, best-corrected visual acuity; OD, oculus dexter; OS, oculus sinister. *p < 0.001.

### Global network topology analysis

**Figure 2:**
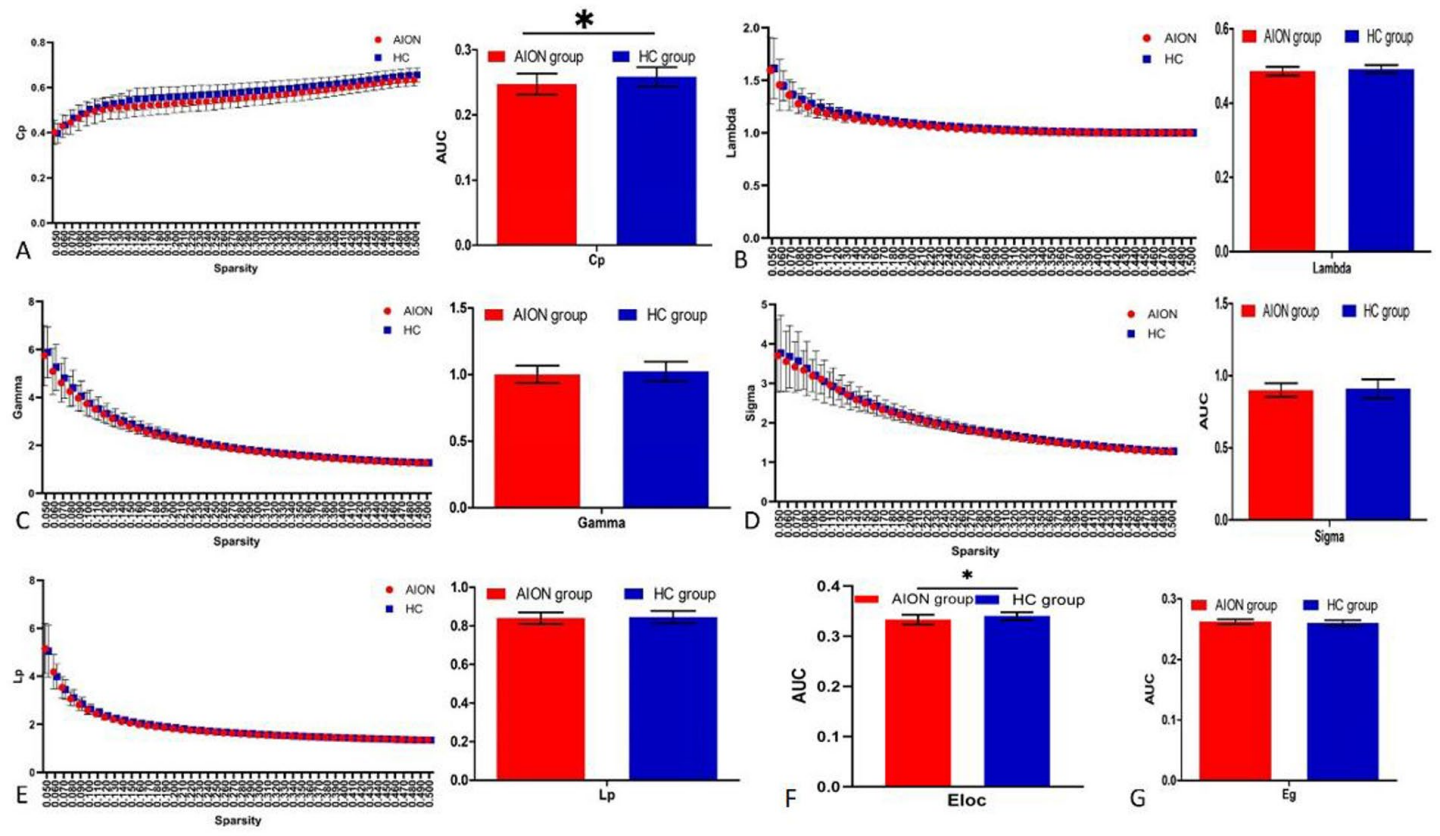
Graphs show that in the defined range of sparsity (0.032 < S < 0.492), both the AION and HC groups exhibited typical features of small-world properties (*γ* = Cpreal/Cprand>1,*λ* = Lpreal/Lprand≈1). The circle and square correspond to the mean value of AION and HCs, respectively, and error bars to the standard error of the subject group in each state. The AUC of small-word properties and network efficiency was shown in histogram graphs (A-F). Cp, clustering coefficient; Lp, characteristic path length; γ, normalized clustering coefficient; λ, normalized shortest path length; Eloc, local efficiency; Eglob, global efficiency; AUC, area under curve; AION, Anterior segment ischemic optic neuropathy; HC, health control.

When we compared the global network indicators of AION patients with the Hc group, we found that the Cp (*p* = 0.0119) and Eloc (*p* = 0.0048) of the functional brain network decreased in AION patients. In contrast, there was no significant change in *γ* (*p* = 0.2403), *λ* (*p* = 0.9189), characteristic path length (Lp) (*p* = 0.5138), small world (*σ*) (*p* = 0.5327) and global efficiency (Eglob) (*p* = 0.8063) in AION patients (Table 4; Figure 2).

**Table 4:** Significant differences in integrated global network parameters between two groups.

	Network parameters	*T-*value	*P-*value
Network Efficiency	E_glob_	1.782	0.8063
	E_loc_	−2.948	0.0048*
Small world	Cp	−2.604	0.0119*
	γ	−1.188	0.2403
	λ	−1.717	0.9189
	Lp	−0.657	0.5138
	σ	−0.628	0.5327

The network efficiency parameters and small-world parameters comparisons in patients with AION and HCs. The symbol “*” denotes *p* < 0.05. (two sample *t*-tests, *p* < 0.05, uncorrected). Cp, clustering coefficient; Lp, characteristic path length; γ, normalized clustering coefficient; λ, normalized characteristic path length; σ, scalar smallworldness; Eglob, global efficiency; Eloc, local efficiency; AION, Anterior segment ischemic optic neuropathy; HC, health control.

### Nodal characteristics of brain functional networks

**Figure 3:**
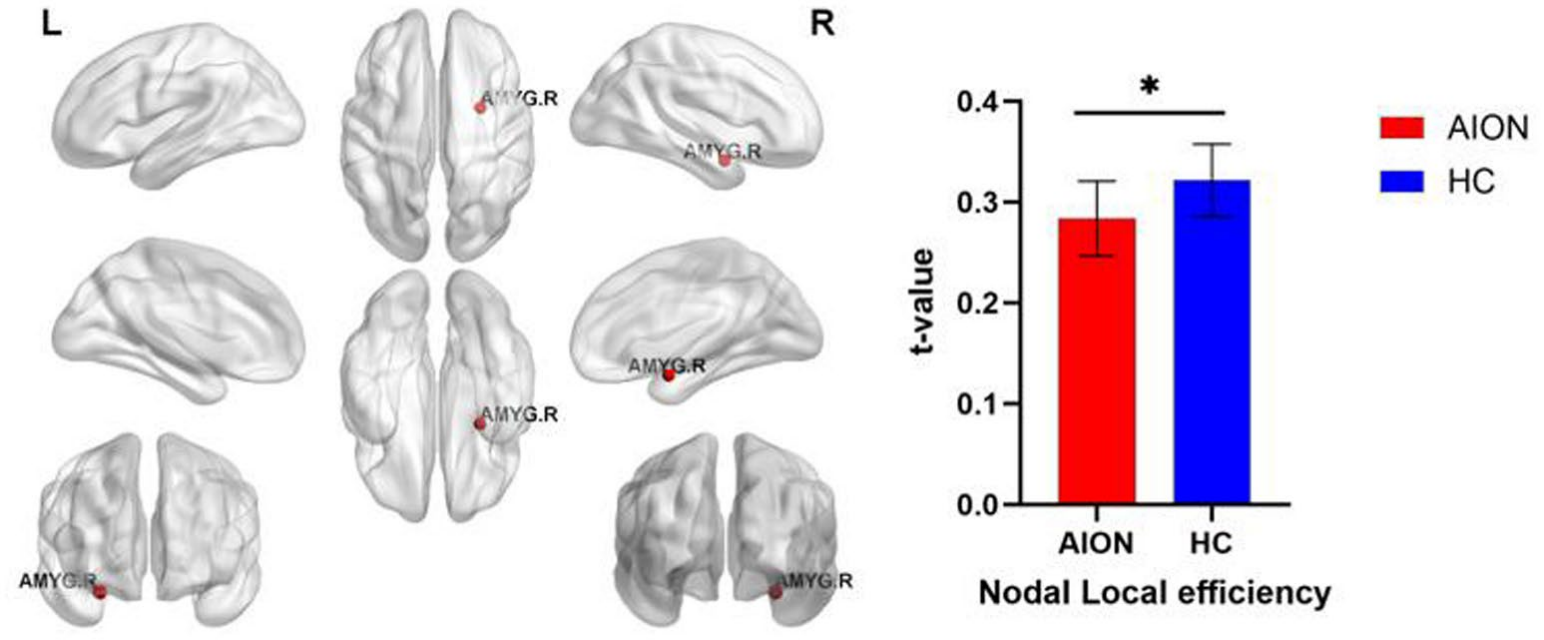
Significant nodal characteristics map the differences between two groups. The AION group had a significant decreased Nodal local efficiency in the right Amygdala. AMYG, Amygdala; AION, Anterior segment ischemic optic neuropathy; HC, health control.

We identified brain regions that exhibited significant between-group differences in at least one nodal metric (*p* < 0.05, Bonferroni-corrected). In comparison to the HC group, the AION group demonstrated significant reductions in Nodal local efficiency in the right Amygdala (Table 5; Figure 3).

**Table 5:** Significant differences in nodal characteristics between AION and HCs.

	Brain regions	*T*-values	*P*-values
AION<HC			
Nodal local efficiency	Right amygdala	−3.7491	0.0004

Bonferroni correction was applied to each nodal characteristic, the *p*-value thresholds for nodal characteristics were 0.05. AION, Anterior segment ischemic optic neuropathy; HC, health control.

### Group differences in functional connectivity

**Figure 4:**
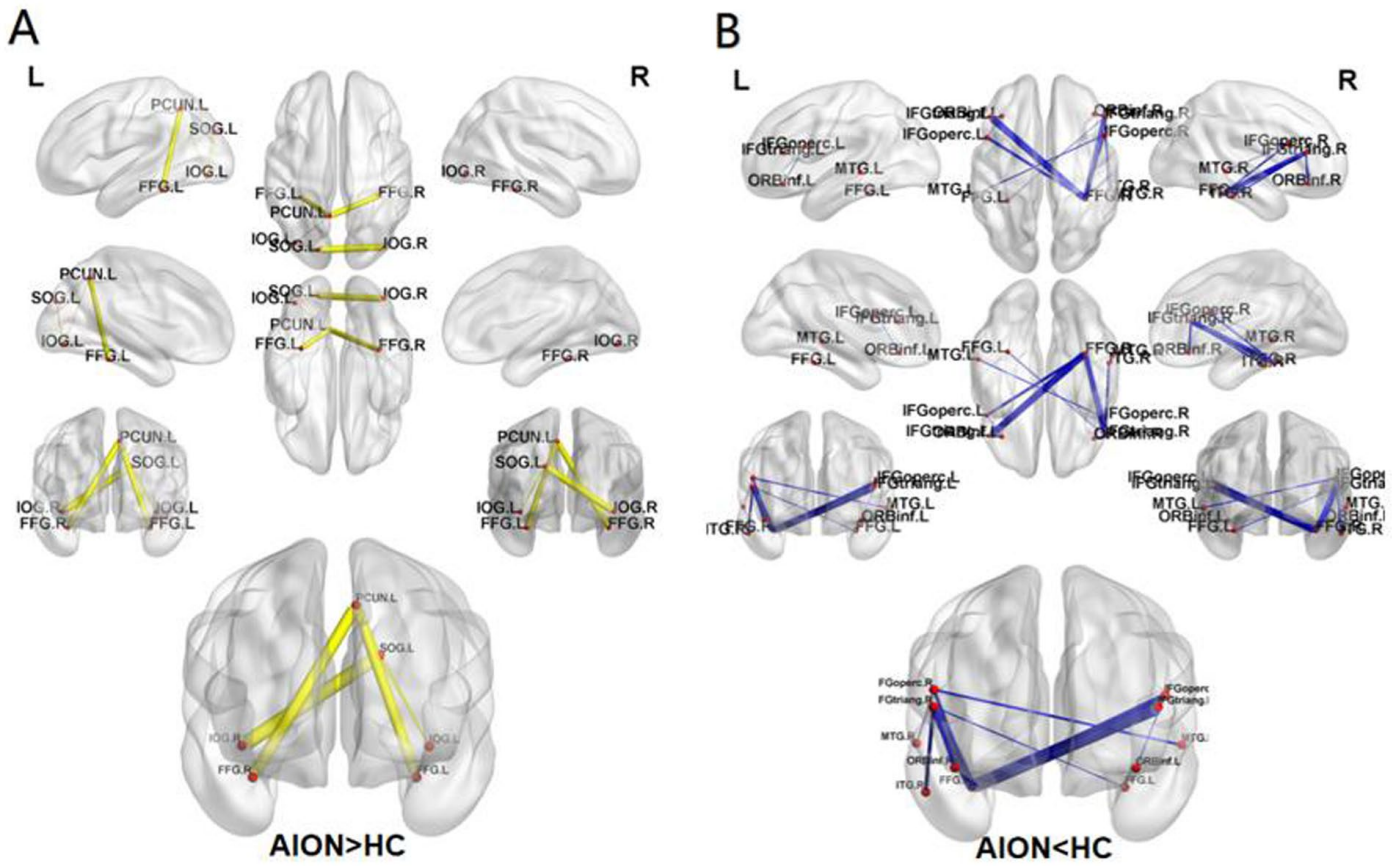
The connected network shows the disrupted functional connections in AION group identified by using NBS (*p* < 0.05; NBS corrected). The increased FC (AION>HC) **(A)**, The decreased FC (AION<HC) **(B)**. SOG, Superior occipital gyrus; IOG, Inferior occipital gyrus; FFG, Fusiform gyrus; PCUN, Precuneus; IFG operc, Inferior frontal gyrus, opercular part; IFG triang, Inferior frontal gyrus, triangular part; ORBinf, Inferior frontal gyrus, orbital part; MTG, Middle temporal gyrus; ITG, Inferior temporal gyrus; NBS, network-based statistics; L, left; R, right; AION, Anterior segment ischemic optic neuropathy; HC, health control.

The NBS method identified a significantly altered network (15 nodes, 15 connections) in the AION group compared to HC group (*p* < 0.05; NBS corrected). Compared with the HC group, the AION group showed increased functional connectivity between the left superior occipital gyrus and the left inferior occipital gyrus, the left superior occipital gyrus and the right inferior occipital gyrus, the left inferior occipital gyrus and the left precuneus, the left fusiform gyrus and the left precuneus, and the right fusiform gyrus and the left precuneus. The functional connectivity between left opercular inferior frontal gyrus and left orbital inferior frontal gyrus, right triangular inferior frontal gyrus and right orbital inferior frontal gyrus, right triangular inferior frontal gyrus and left fusiform gyrus, left opercular inferior frontal gyrus and right fusiform gyrus, right opercular inferior frontal gyrus and right fusiform gyrus, left triangular inferior frontal gyrus and right fusiform gyrus, right triangular inferior frontal gyrus and right fusiform gyrus, right opercular inferior frontal gyrus and left middle temporal gyrus, right opercular inferior frontal gyrus and right middle temporal gyrus, right triangular inferior frontal gyrus and right inferior temporal gyrus were weakened (Table 6; Figure 4).

**Table 6:** Significantly altered functional connectivities in AION patients compared with HCs.

Label	Brain region 1	Brain region 2	*T*-value	*P*-value
AION>HC	SOG.L	IOG.L	3.5614	0.0008
	SOG.L	IOG.R	3.8936	0.0003
	IOG.L	PCUN.L	3.5448	0.0008
	FFG.L	PCUN.L	3.7983	0.0004
	FFG.R	PCUN.L	3.8346	0.0003
AION<HC	IFG operc.L	ORBinf.L	−3.6407	0.0006
	IFG triang.R	ORBinf.R	−3.9990	0.0002
	IFG triang.R	FFG.L	−3.6854	0.0005
	IFG operc.L	FFG.R	−4.2078	0.0001
	IFG operc.R	FFG.R	−3.9082	0.0003
	IFG triang.L	FFG.R	−5.1869	3.57E-06
	IFG triang.R	FFG.R	−4.7629	1.57E-05
	IFG operc.R	MTG.L	−3.8276	0.0003
	IFG operc.R	MTG.R	−3.6132	0.0007
	IFG triang.R	ITG.R	−3.9392	0.0002

NBS method identified a significantly altered network (15 nodes and 15 connections) in AION group relative to HCs (*P* < 0.05; NBS corrected). NBS, network-based statistics; L, left; R, right; AION, Anterior segment ischemic optic neuropathy; HC, health control.

### Relationships between network properties and clinical variables in the AION group

In this study, we analyzed the correlation between global network parameters, node parameters and clinical data such as gender, course of disease, visual acuity and intraocular pressure of AION patients. There was no significant correlation between the global and node parameters of brain network and gender, course of disease, visual acuity and intraocular pressure in AION patients (*p* > 0.05).

## Discussion

In this study, fMRI in conjunction with graph theory was utilized to investigate the topological alterations in functional brain networks among AION patients. The main outcomes of our research are as follows: (1) Significantly decreased Cp (*p* = 0.0119) and Eloc (*p* = 0.0048) were observed in the AION group compared to the HC group. (2) Notably, the AION group exhibited pronounced reductions in Nodal local efficiency within the right Amygdala relative to the HC group. (3) By employing the NBS method, a significantly altered network comprising 15 nodes and 15 connections was identified in the AION group compared to HCs. These collective findings provide novel insights into the brain’s functional connectome, offering valuable targets and biological markers for potential clinical interventions for AION.

In the framework of graph theory, there are three types of networks: regular, small-world, and random network, and these networks are judged by cluster coefficient and characteristic path length (Guan et al., 2022). Combining the advantages of regular and random networks, the small-world network has a higher clustering coefficient, which is favorable for local specialized processing, and a shorter characteristic path length, which is favorable for global distributed processing (Bullmore and Sporns, 2009; Bassett and Bullmore, 2017). In the current study, both the AION and HC groups exhibited comparable small-world characteristics in the brain’s functional network. Nevertheless, AION patients demonstrated reduced Cp and Eloc in comparison to HCs. Cp denotes the propensity for neighboring nodes of a specific node to be interconnected, reflecting the degree of local connectivity (Rubinov and Sporns, 2010). Consequently, reduced Cp and Eloc imply diminished local connectivity within functional networks, leading to decreased efficiency in information transfer among interconnected regions in AION patients. Global efficiency measures the ability of parallel information propagation within a network. High global efficiency ensures effective integrity and rapid information propagation between and across remote brain regions that are believed to constitute the basis of cognitive processing (Sporns and Zwi, 2004). The decreased global efficiency may reflect disrupted neuronal integration among distributed regions as demonstrated by numerous disruptions in intermodule connectivity, which play a critical role in coordinating neural activity over the entire brain (Wang et al., 2015). The decrease in global efficiency also reflects the shift of brain functional connectivity to randomization (that is, loss of isolation, which is conducive to global integration) in patients with AION. Separated measurements showed that special processing occurred in densely connected brain regions (Wig, 2017). Smaller isolation or special topology may be beneficial to network function (Chan et al., 2014). Integration measures the ability to combine specialized information between distributed brain regions. The preference for global integration may promote the efficiency of global communications and integrate distributed information. On the whole, less separation and preference for integration lead to the randomization of the network. The randomization also conforms to the characteristic that the degree distribution is thick tail. Therefore, it can be predicted that the brain functional connectivity of AION patients has more topological randomness than that of healthy controls. In alignment with the global topology, we investigated the node characteristics of the functional brain connectivity in AION patients, unveiling notable deviations from the overall network structure. These node attributes serve as role indicators for facilitating the integration and transmission of information within the network. Notably, the AION group exhibited significant decreases in Nodal local efficiency in the right Amygdala compared to the HC group. The superior colliculus-thalamic occipital tubercle-amygdala pathway is one of the visual subcortical pathways in the mammalian brain. The amygdala is the last station of this pathway and the most widely studied brain structure in this pathway. It is mainly responsible for the information processing of emotions (mainly negative emotions, such as fear), especially under consciousness (Wang et al., 2022; Phelps and LeDoux, 2005; Whalen et al., 2004). In the early perception process, the amygdala receives information about emotions, and then projects to the sensory cortex to further regulate attention and perception. When new emotional stimuli appear, the excitement of the visual cortex will be enhanced, and this enhanced excitement of the visual cortex is related to the excitement of the amygdala (Meier et al., 2021). In this study, the nodal local efficiency of amygdala in AION patients was lower than that in healthy controls, which may indicate that AION patients have attention and cognitive impairment. The NBS method identified a significantly altered network (15 nodes, 15 connections) in the AION group compared to HC group (*p* < 0.05; NBS corrected). These nodes are mainly distributed in the frontal lobe, occipital lobe, temporal lobe and superior parietal lobule. The frontal lobe, located anterior to the central sulcus and above the lateral fissure, is the most complex part of the brain. The IFG has been associated with emotional and cognitive empathy (Shamay-Tsoory et al., 2009), offer quality (de Berker et al., 2019), and attentional control (Hampshire et al., 2010). Previous studies have found that a number of optic disease lead to the IFG dysfunction, including optic neuritis (Sun et al., 2021), anisometropic amblyopia (Lin et al., 2012), strabismus and amblyopia (Shao et al., 2019), and primary angle-closure glaucoma (Chen et al., 2019). The temporal pole is part of the parahippocampal area and is a transitional region from the surrounding cortex to the neocortex. The ITG in the last stage of the ventral visual pathway and are important components of the occipitotemporal network. The inferior temporal cortex is known to play a critical role in the visual representation of form and color, and the temporal pole is also involved in processing visual stimuli (Gross, 1992; Skipper et al., 2011). The decrease of brain functional connectivity in AION patients reflects the decrease of the ability of the brain to process information cooperatively, which may represent the functional decompensation caused by the disease. In this study we found that the functional connection between the frontal lobe and temporal lobe was weakened in AION patients, which may reflect the loss of eye movement, decreased cognitive function, and persistent dysfunction of neural networks in these patients.

In addition, the visual cortex is the primary cortical region of the brain that receives, integrates, and processes visual information relayed from the retinas. It is in the occipital lobe of the primary cerebral cortex, which is in the most posterior region of the brain. As part of the primary visual network, the fusiform gyrus is an important link in the human ventral visual information processing pathway that processes color information and facial expression perception (Radua et al., 2010; Müller et al., 2018). The precuneus (PCUN) is mainly associated with visuospatial exploration and memory of visual images (Cavanna and Trimble, 2006; Fairhall and Ishai, 2008). The enhancement of brain functional network connectivity in AION patients may reflect the compensatory enhancement of brain function caused by the disease. The results showed that the enhancement of functional connectivity between occipital lobes and between occipital lobes and superior parietal lobules may reflect the compensation of visual function in AION patients.

This study has several limitations. The stability of the results obtained could have been impacted by the relatively small sample size. Additionally, AION typically presents unilaterally, with the possibility of the other eye being affected years later. However, due to ineffective treatment, patients often seek help from multiple clinics. Consequently, some patients arriving at our hospital already exhibit AION in both eyes, making it challenging to recruit individuals with only one affected eye. The involvement of different eyes may influence the results of AION brain networks. In future studies, we plan to conduct a more in-depth analysis of the differences in AION brain networks between affected eyes.

## Conclusion

Patients with AION exhibited topological abnormalities in human brain connectivity. Specifically, there was a reduction in Cp and Eloc within the AION group compared to the HC group. The atypical node centralities and functional connections in AION patients were predominantly located in the prefrontal lobe, temporal lobe, and parietal lobe. These findings provide valuable insights into the neural mechanisms underlying visual impairment, disrupted emotion regulation, and cognitive deficits in AION individuals.

## Data Availability

The raw data supporting the conclusions of this article will be made available by the authors, without undue reservation.

## Footnotes

^1^http://www.nitrc.org/projects/gretna/

^4^http://www.nitrc.org/projects/nbs/
